# Hepatic involvement in Wegener's granulomatosis: a case report

**DOI:** 10.1186/1752-1947-4-9

**Published:** 2010-01-14

**Authors:** Constantin Goritsas, Nicolas P Paissios, Rodoula Trigidou, Joanna Delladetsima

**Affiliations:** 1 Department of Internal Medicine, Sotiria General Hospital, 152 Mesogeion Avenue, Athens, 11527, Greece; 2 Department of Pathology, Sotiria General Hospital, 152 Mesogeion Avenue, Athens, 11527, Greece; 3 Department of Pathology, Laiko University Hospital, Ag Thoma, Athens, 11527, Greece

## Abstract

**Introduction:**

We report the case of a 58-year-old Caucasian Greek man who presented with dry cough, fever, bilateral alveolar infiltrates and acute hepatitis.

**Case presentation:**

After a lung biopsy, the patient was diagnosed with Wegener's granulomatosis. The diagnosis was supported by the presence of anti-proteinase-3 anti-neutrophil cytoplasmic antibodies. A liver biopsy demonstrated the presence of mild non-specific lobular hepatitis and periodic acid-Schiff positive Lafora-like inclusions in a large number of his liver cells. The patient was treated with prednisone and cyclophosphamide, which was followed by subsequent remissions of chest X-ray findings and liver function studies.

**Conclusion:**

What makes this case worth reporting is the coexistence of liver inflammation with a biochemical profile of severe anicteric non-viral, non-drug induced hepatitis coinciding with the diagnosis of Wegener's granulomatosis. Our paper may be the first report of hepatic involvement in a patient diagnosed with Wegener's granulomatosis. The aetiological link between the two diseases is supported by the reversion of hepatitis after the immunosuppression of Wegener's granulomatosis. We favor the hypothesis that hepatic vasculitis may be the cause of acute hepatocellular necrosis.

## Introduction

Wegener's granulomatosis is an anti-neutrophil cytoplasmic autoantibody (ANCA)-associated small vessel systemic vasculitis characterized primarily by necrotizing granulomatosis of the upper and lower respiratory tract and by glomerulonephritis. We report the case of a 58-year-old man with symptoms of Wegener's granulomatosis who developed acute anicteric hepatitis.

## Case presentation

A 58-year-old Caucasian man of Greek origin and nationality was transferred to our hospital's Internal Medicine Department due to a marked elevation of his hepatic enzymes. He had a long history of rhinitis considered to be of allergic origin and with an epidermal sensitivity test that was positive to parietaria species. He had no history of alcohol abuse.

Nine months before he was admitted to our hospital, he developed an acute hearing loss in his right ear (treated without improvement with dimenhydrinate and pyridoxine by his otolaryngologist), which was followed shortly after by a worsening dry cough. Eight weeks before our evaluation, he reported fatigue, loss of appetite and weight loss of about 4 kg to 5 kg. In the last three weeks of that same period, his temperature rose to 38.8°C and he was treated without improvement with oral antibiotics (cefprozil and clarithromycin) by his local physician. Due to the persistence of his fever, after being treated with antibiotics for seven days he was referred by his local physician to the pneumonology department of our hospital. At that time, he was afebrile. Chest radiography revealed the presence of bilateral alveolar infiltrates, while liver function tests were within normal range. Bronchoscopy was negative for structural abnormalities, and culture of bronchoalveolar lavage fluid was negative. A chest computed tomography (CT) scan was performed in which the presence of multiple lung masses (nodular opacities) was noted. The result of a Mantoux skin test was negative and a complete immunologic serologic profile was pending when the patient was transferred to our department because of the elevation of his hepatic enzymes, starting a week after his admission.

On examination, his temperature was 36.8°C, heart rate was 68 beats/min, respiratory rate was 16 breaths/min, and blood pressure was 140/70 mmHg. His lungs were clear to auscultation bilaterally. His abdomen was neither tender nor distended, and his liver was palpable 4 cm below the costal margin. The findings on the rest of the examination procedures were normal, as was his echocardiogram. Laboratory investigation showed the following data: white blood cell count, 10.7 × 10^9^/L (with 66% neutrophils); hemoglobin, 11.4 g/dL; mean cell volume, 93.9fl; platelet count, 437 × 10^9^/L; erythrocyte sedimentation rate, 92 mm/1 h; C-reactive protein, 8.8 mg/dl; creatinine, 0.9 mg/dL; serum urea nitrogen, 34 mg/dL; aspartate aminotransferase, 781 U/L; alanine aminotransferase, 512 U/L; alkaline phosphatase, 322 U/L; albumin, 3.1 g/dL; α_1_-antitrypsin, 184 mg/dL; IgG, 1980 mg/dl; IgA, 413 mg/dl; and IgM, 55.6 mg/dl. A urine analysis was negative for glycosuria and proteinuria, while urine microscopy revealed 3 to 5 red blood cells per high power field and the presence of occasional dysmorphic red blood cells.

The tests for hepatitis B, hepatitis C, hepatitis A and human immunodeficiency viruses all returned negative results. The results from the immunologic assays denoted the presence of ANCA against proteinase 3, which was confirmed by both immunofluorescence assay and Elisa anti-proteinase 3 antibodies. At the same time, tests for antinuclear antibodies, anti-smooth muscle antibodies and anti-liver kidney microsomal antibodies were all negative. An upper abdomen CT scan also yielded normal results.

**Figure 1:**
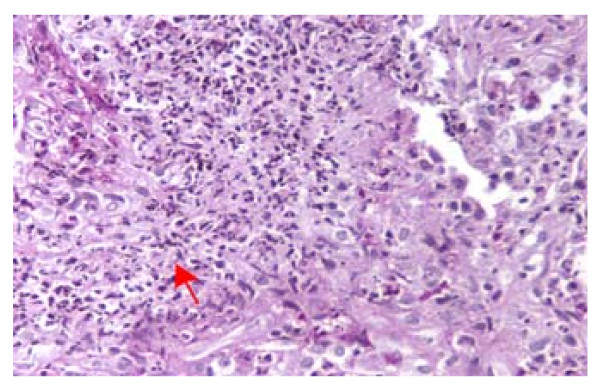
**Lung biopsy (fine needle biopsy), periodic acid Schiff stain ×400 magnification**.

**Figure 2:**
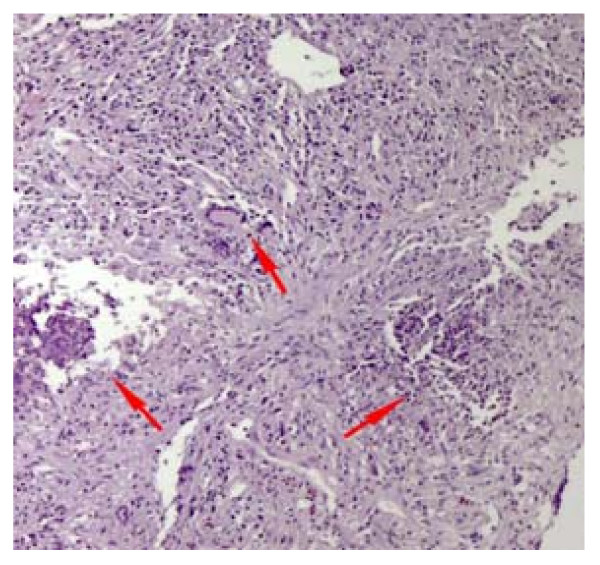
**Lung biopsy (fine needle biopsy), hematoxylin and eosin stain ×400 magnification**.

**Figure 3:**
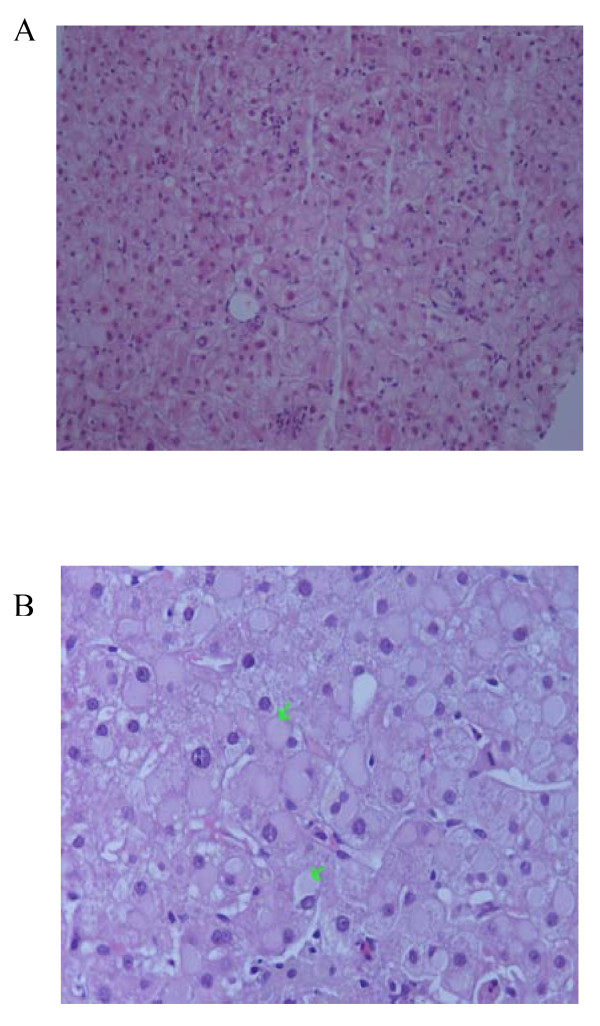
**(A) Liver biopsy showing focal necrosis with neutrophils and sinusoidal reaction, hematoxylin and eosin stain ×400 magnification [R1]**. **(B) **Liver biopsy showing Lafora body-like inclusions, hematoxylin and eosin stain ×400 magnification [R2].

A biopsy of our patient's nasal mucosa did not reveal any changes that were compatible with necrotizing vasculitis. A complete ophthalmologic examination was also performed and was negative for ocular abnormalities. Our patient's liver function tests kept worsening and his transaminase levels reached a 15-fold increase within a week following admission. However, his bilirubin remained within normal values (1.2 mg/dl). A fine needle biopsy of his pulmonary lesions was obtained and the histological findings were compatible with Wegener's granulomatosis. The characteristic pathological features were irregular areas of necrosis surrounded by inflammatory granulation tissue (Figure 1) and neutrophilic microabscesses were surrounded by a granulomatous reaction with an occasional multinucleate Langhan's giant cell (Figure 2). A liver biopsy was also performed, and histological evaluation revealed mild non-specific lobular hepatitis characterized by randomly distributed focal hepatocellular necrosis with collections of neutrophils and lymphocytes. A few apoptotic cells and moderate sinusoidal reaction were also observed. A large number of liver cells exhibited periodic acid Schiff-positive Lafora-like inclusions. Occasional portal tracts showed mild leucocytic infiltrations (Figure 3).

**Figure 4:**
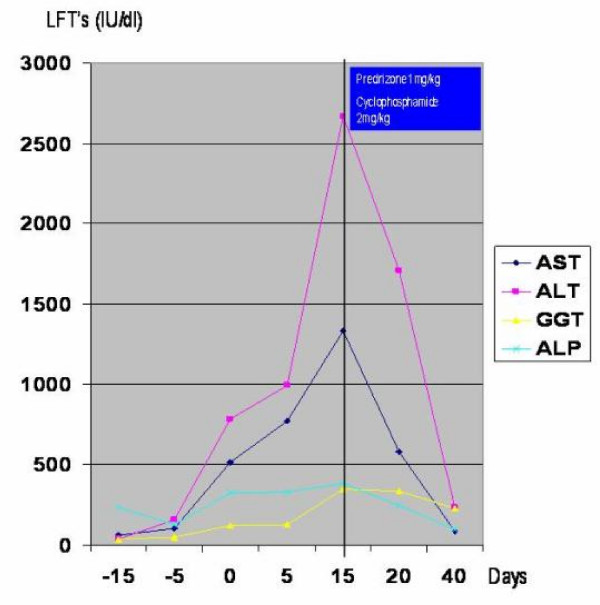
**Liver function tests in the course of time (0 on the X axis stands for the first day of admission to our department)**.

Our patient later received a combined treatment of 1 mg/kg/day prednisone and 2 mg/kg/day of cyclophosphamide. Our patient's status gradually improved with a remission of both the chest X-ray findings and the liver function studies 25 days after the initiation of treatment (Figure 4). After all signs of active disease had disappeared, a gradual tapering of corticosteroid and cyclophosphamide therapy was initiated. Eighteen months after his hospitalization, the patient is free of symptoms, with normal results for liver function tests and without any sign of relapse.

## Discussion

Our patient had a long history of rhinitis that may represent a slowly progressive mild Wegener's granulomatosis. The main onset of symptoms was the acute hearing loss, which reports mention as present in about 10% of patients at the time of their presentation and in 40% overall during the course of their illness. The diagnosis of Wegener's granulomatosis was based on the clinical manifestations, imaging studies, and pathognomonic tissue abnormalities from our patient's lung biopsy. The diagnosis was further supported by the presence of c-ANCAs, mild leukocytosis and normocytic normochromic anemia in our patient.

What was surprising, however, and what makes this case worth reporting, was the coexistence of liver inflammation with a biochemical profile of severe anicteric hepatitis at the time of Wegener's granulomatosis diagnosis. Drug-induced liver damage was highly unlikely because liver dysfunction and the elevation of hepatic enzymes persisted after the discontinuation of antibiotics. A viral hepatitis was ruled out considering that serologies for acute HBV, HAV and HCV infections were repeatedly negative when checked at different times, that the possibility of ischemic hepatitis in a patient without previous heart condition history was nil, and that our patient had hemodynamic stability and a perfectly normal echocardiogram.

Autoimmune hepatitis (AIH) cannot be ruled out as well, since 10% of these cases may present with negative antinuclear antibodies, anti-smooth muscle antibodies, and anti-liver kidney microsomal antibodies. According to the revised criteria and scoring system by the International AIH Group [1], our patient's AIH score was 11+, a fact that initially made this diagnosis probable. Furthermore, ANCAs are positive in 65% to 95% of patients with AIH. However, they are usually of the perinuclear type and the histological findings from the liver biopsy in our patient were non-specific.

In patients with systemic vasculitis syndromes, clinical manifestations in the liver are not frequent, although a case series notes that liver arteritis is not an infrequent finding, especially in patients with polyarteritis nodosa [2-4] or systemic lupus erythematosus [5]. The most common hepatic complications in patients with polyarteritis nodosa as reported in the literature [3,4] are hepatic infarction or aneurysm rapture, acute liver failure [6], ischemic cholangitis [7], and nodular regenerative hyperplasia [7,8]. Necrotizing arteritis of the liver was found in between 10% and 21% of patients with systemic lupus erythematosus in an autopsy series [5]. However, these findings are not related to hepatic clinical manifestations. In Wegener's granulomatosis, although virtually any organ can be involved with vasculitis, granulomas or both, gastrointestinal involvement is very uncommon and the liver is extremely rarely affected [9]. Only isolated cases associated with primary biliary cirrhosis and hepatic granulomas are reported. Shah *et al. *[10], reported a case of Wegener's granulomatosis with perivascular and periportal granuloma with Langhan's giant cells. Boissy *et al. *[11] reported a case of Wegener's granulomatosis in which portal inflammatory granuloma and vasculitis with portal tract fibrosis and venous lesions were found. Zen *et al. *[12] reported a case of incomplete septal cirrhosis associated with Wegener's granulomatosis and Sjögern's syndrome. A very rare form of hepatotoxicity was reported only twice in the literature [13,14].

Cyclophosphamide-induced noncaseating granulomatous hepatitis in Wegener's granulomatosis was also ruled out from the start in this case, because hepatitis was apparent well before the initiation of Wegener's granulomatosis treatment with cyclophosphamide. In our case, neither vasculitis of the portal tracts nor granulomas that would have revealed a possible necrotizing angiitis (Wegener's granulomatosis type) of the liver was detected. Nevertheless, this possibility cannot be excluded, since only seven portal tracts were examined. The histological findings of mild hepatic necrosis in acinar zones 3 and 2 with concomitant mild inflammation of predominantly neutrophils and mononuclear cells with portal tracts and the nearly normal periportal parenchyma are compatible with vascular damage resulting in the hypoperfusion of the liver. This hypothesis is reinforced by the immediate response of liver function tests after immunosuppressive therapy with prednisone and cyclophosphamide was initiated.

The histological finding of ground-glass inclusions within hepatocytes is common in HBV infection and has also been reported in drug-induced liver damage (cyanamide, disulfiram), Lafora's disease (myoclonus epilepsy), type IV glycogenosis, fibrinogen storage disease, and acute veno-occlusive disease [15]. There have also been several reports of immunosuppressed patients on numerous medications with ground-glass hepatocellular inclusions [16]. Our patient had normal serum levels of α_1_-antithrypsin, was HBV seronegative, had no history of drug abuse, and did not demonstrate clinical findings of myoclonus epilepsy or seizures under a normal electroencephalogram test.

Recently, Lefkowitch *et al. *[17] suggested that ground-glass inclusions within hepatocytes are the result of disturbed glycogen metabolism. Whether this cytoplasmic change in our patient was an incidental finding due to his disturbed glycogen metabolism or was related to the liver involvement of Wegener's granulomatosis cannot be ascertained. In this case, liver biopsy showed non-specific hepatitis with mild lobular activity. The absence of histological findings compatible with Wegener's granulomatosis could be attributed to sampling, since liver damage in Wegener's granulomatosis is expected to be focal and not diffused.

## Conclusion

To the best of our knowledge, this report presents one of the first cases of hepatic involvement in a Wegener's granulomatosis patient. The aetiological link between the two diseases is supported by the reversion of hepatitis after the immunosuppression of Wegener's granulomatosis, and we favor the hypothesis that hepatic vasculitis may be the cause of acute hepatocellular necrosis. The simultaneous finding of Lafora body-like inclusions in our patient's hepatocytes makes the case even more intriguing, even when an incidental coincidence cannot be ruled out.

## Consent

Written informed consent was obtained from the patient for publication of this case report and any accompanying images. A copy of the written consent is available for review by the Editor-in-Chief of this journal.

## Competing interests

The authors declare that they have no competing interests.

## Authors' contributions

GC and NP were the patient's physicians. RT performed the histological examination of the nasal mucosa and the lung biopsy. JD and RT performed the histological examination of the liver biopsy.

All authors have read and approved the final manuscript
